# Is the BioSentry Tract Sealant System Effective in Reducing the Pneumothorax Rate After CT-Guided Transthoracic Lung Biopsy With an 18G Biopsy Needle?

**DOI:** 10.7759/cureus.71078

**Published:** 2024-10-08

**Authors:** Kiyon Naser-Tavakolian, Michael Clifton, Amir Mahmoud, Amit Gupta

**Affiliations:** 1 Radiology, Stony Brook University Hospital, Stony Brook, USA; 2 Interventional Radiology, Columbia University, New York, USA; 3 Interventional Radiology, University of Minnesota, Minneapolis, USA; 4 Radiology, University of California Davis Medical Center, Davis, USA; 5 Interventional Radiology, The Ohio State University Wexner Medical Center, Columbus, USA

**Keywords:** biopsy needle, chest tube, lung biopsy, pleural sealant device, pneumothorax

## Abstract

Introduction: This study evaluated whether the BioSentry Tract Sealant System (BTS) (Merit Medical Systems, South Jordan, Utah) reduces pneumothorax rates in CT-guided lung biopsies (CTLB) using an 18G biopsy/17G introducer needle.

Materials and methods: A total of 272 patients underwent CTLB between January 2015 and February 2019 using a 17G/18G system. BTS was used in 125 patients, while no pleural sealant was used in 147. The variables assessed included pneumothorax on post-biopsy CT images or chest X-ray, chest tube insertion, lesion size, pleura-to-lesion distance, and the presence of emphysema. The two groups were compared using T-tests with a significance level of 0.05.

Results: In the BTS group, 23 subjects (18%) had a pneumothorax compared to 38 (26%) in the control group (p < 0.05). Four patients (17%) in the BTS group required a chest tube, compared to three patients (8%) in the control group (p = 0.28). The mean lesion size in patients with a pneumothorax was 1.62 cm in the BTS group and 2.44 cm in the control group (p = 0.27). The mean pleura-to-lesion distance in those with pneumothorax was 2.23 cm in the BTS group and 1.52 cm in the control group (p = 0.17). Emphysema was present in 47% of patients in the BTS group and 30% in the control group (p < 0.05).

Conclusion: BTS reduced pneumothorax rates in CTLB patients using a 17G/18G system, despite a higher proportion of emphysema patients in the BTS group. No significant difference in the rate of chest tube insertion was observed with or without BTS, which contrasts with previous studies where 19G/20G biopsy systems were used.

## Introduction

Pneumothorax is the most common complication of percutaneous CT-guided lung biopsy [1]. The rate of pneumothorax occurrence after CT-guided lung biopsies is approximately 15-25%, with a higher incidence seen in patients with risk factors such as emphysema [1-5]. Additionally, chest tube placement may be required if the pneumothorax increases in size or causes symptoms such as dyspnea. Several techniques have been described to decrease pneumothorax rates post-biopsy, including blood patch, saline, collagen, gelatin sponge slurry, hydrogel sealant, and aspiration with retraction of the needle [6-17]. In 2012, a biodegradable pleural plug called the BioSentry Tract Sealant System (BTS) (Merit Medical Systems, South Jordan, Utah) was approved by the US Food and Drug Administration (FDA) to reduce pneumothorax and chest tube insertion rates after percutaneous lung biopsy using a 19G introducer needle and a 20G core biopsy needle. However, many physicians prefer a larger 17G introducer needle and an 18G core biopsy needle (17G/18G system). This retrospective study aims to assess the efficacy of BTS in reducing pneumothorax and chest tube insertion rates when a 17G/18G system is used. This article was previously presented as a meeting abstract at the Society of Interventional Radiology Annual Meeting on March 29, 2020, and the abstract was published in the *Journal of Vascular and Interventional Radiology* supplement issue for March 2020.

## Materials and methods

We conducted a retrospective assessment of all pulmonary lung nodule biopsies from 2015 to 2019 utilizing 17G/18G coaxial systems and evaluated whether BTS reduced pneumothorax rates when using a larger bore coaxial biopsy system than its original study. This was an Institutional Review Board (IRB)-approved retrospective study in which 272 patients (mean age 68.9) underwent CT-guided lung biopsies between 2015 and 2019 using a 17G/18G coaxial biopsy/introducer system. BTS was used in 125 patients, while 147 patients received no pleural sealant. The inclusion criteria were adults (>18 years old) who underwent CT-guided lung biopsies between 2015 and 2019. The only exclusion criterion was pediatric patients. The primary variables of interest were the presence of pneumothorax on the post-procedure CT scan and the need for chest tube placement to manage the pneumothorax. Additional variables considered were lesion size, pleura-to-lesion distance, the presence of emphysema, and perilesional/intraparenchymal hemorrhage. The variables were compared between the two groups using T-tests and chi-squared tests, with the significance level set at 0.05.

An attending interventional radiologist reviewed the clinical history and chest CT scan (with or without PET imaging) prior to each biopsy to determine the appropriateness and feasibility of a transthoracic lung biopsy. Typically, lesions close to the hilum were referred to interventional pulmonology for transbronchial biopsy. Prior chest CT imaging reports were reviewed to identify whether patients had emphysema, and this was documented under the patient characteristics.

Patients underwent lung biopsy with local anesthesia, sedation, or general anesthesia, depending on several factors such as medical comorbidities, estimated procedure time, and lesion location. The shortest distance between the pleura and the lesion was chosen whenever possible. However, other factors, such as patient comfort, critical structures between the skin and the lesion, and bullae, were also considered when choosing the needle trajectory.

All biopsies were performed using at least a 16-slice CT scanner. The scanners were not equipped with CT fluoroscopy. All biopsies were performed by the same attending interventional radiologist, who has more than 25 years of experience. When conscious sedation was used, it was typically administered before the initial CT images to allow for its effect on respiratory excursions before marking the needle skin entry site. After local anesthesia, a 17G coaxial introducer needle was advanced toward the chosen lesion with intermittent CT imaging. Once in position, multiple core biopsies were obtained using 18G core biopsy needles (Biopince by Argon Medical, Texas, or Corvocet by Merit Medical, Utah). The pathology technologist or physician performed Rapid On-Site Evaluation (ROSE) for each biopsy procedure. After obtaining the core biopsy specimens, a limited CT of the chest was obtained to assess for post-procedural complications, particularly pneumothorax and parenchymal hemorrhage. The attending interventional radiologist decided whether to use BTS based on their experience. Typically, patients with significant emphysema and a history of smoking received BTS. If a pneumothorax was present but the introducer needle tip was still in the lung parenchyma, BTS could still be used per the manufacturer’s instructions. Biosentry was utilized according to its standardized indication for use, deployment, and mechanism of action. The Biosentry device uses desiccated polyethylene glycol hydrogel that deploys as a solid cylinder (2.5 cm in length by 0.1 cm in diameter) and self-expands upon contact with moist tissue to fill the void created by the biopsy needle.

Once the procedure was completed, all patients were monitored in the radiology post-procedural patient holding area. A follow-up chest X-ray was obtained one hour after the procedure if a pneumothorax was noted on the post-procedure CT. Patients could be discharged after approximately two hours if no signs or symptoms of complications were present, or if the pneumothorax was stable or improving. If a post-procedural pneumothorax or a large amount of perilesional hemorrhage was noted on the post-procedure CT, serial assessments with chest X-rays were used for hemodynamically stable patients. Despite monitoring, the development of symptoms or an increasing pneumothorax size was treated with the placement of an 8- to 10-French chest tube (Cook Pigtail Catheter) connected to suction. Our practice uses pigtail catheters for large pneumothoraces instead of larger bore chest tubes, as they have similar efficacy and fewer complications upon insertion [18]. Hemodynamically stable patients with a chest tube were monitored/admitted and returned to interventional radiology after improved chest X-rays for chest tube removal.

## Results

A total of 272 pulmonary lesion biopsies were analyzed retrospectively. The average age of the population was 68.9 years. There were 69 male patients and 78 female patients who underwent CT-guided lung biopsy. Of these, 125 patients received BioSentry sealant post-procedurally. No significant difference was noted in the age of patients between the two groups. There were significantly fewer males in the BioSentry group, but no difference in the number of females between the groups. The average lesion size was 2.63 cm overall. The average lesion size was smaller in the BTS group compared to the no-sealant group (1.92 cm versus 2.63 cm, p < 0.05). There was no significant difference in pleura-to-lesion distance between patients with pneumothoraces and those without in both groups.

In the no-sealant group, patients who developed a pneumothorax had an average lesion size of 2.44 cm (±3.46 cm). In the BTS group, patients who developed a pneumothorax had an average lesion size of 1.62 cm (±1.12 cm). There was no significant difference in average lesion size in patients with pneumothoraces between the two groups (p = 0.27). Similarly, there was no significant difference in pleura-to-lesion distance in patients with pneumothoraces between the BTS and no-sealant groups (2.33 cm and 1.52 cm, respectively, p = 0.17). Additionally, there was no significant difference in pleura-to-lesion distance in patients without pneumothoraces between the BTS and no-sealant groups (1.50 cm and 1.38 cm, respectively, p = 0.31).

The BioSentry cohort had a higher incidence of emphysema (47% vs. 30%) with a significantly lower pneumothorax rate noted on post-procedure CT. However, the deployment of BioSentry did not reduce the overall incidence of chest tube insertion between the two groups, and there was no significant difference in chest tube insertion rates. These findings are outlined in Table 1.

**Table 1:** No Sealant Versus BioSentry Tract Sealant Significance level was designated as p < 0.05 and was determined using two-sample T-tests and chi-squared tests.

Variables	No Sealant		BioSentry			
	Value	SD	Value	SD	T-value	p-value
Average age	68.95		70.05			
Total number of cases	147		125			
Male patients	69		38			<0.05
Female patients	78		87			
Presence of emphysema (%)	30%	4	47%	4	34.9	<0.0001
Pneumothoraces	38		23			
Pneumothorax rate	0.26	0.04	0.18	0.03	18.3	<0.0001
Chest tube insertions	3.00		4.00			
Chest tube insertion rate in those with pneumothoraces	0.08	0.27	0.17	0.38	-0.09	0.28
Average lesion size (cm)	2.63	2.50	1.92	1.40	2.78	0.0058
Average lesion size in those with pneumothorax (cm)	2.44	3.46	1.62	1.12	1.09	0.27
Average pleura to lesion distance with pneumothorax (cm)	1.52	1.81	2.23	2.23	1.36	0.17
Average pleura to lesion distance without pneumothorax (cm)	1.38	1.77	1.60	1.50	1.01	0.31

## Discussion

Recognizing that percutaneous pulmonary lesion biopsy is a common procedure performed by interventional radiology, this study utilized a retrospective analysis to determine whether post-procedural sealant deployment decreased pneumothorax and chest tube insertion rates when using a larger bore introducer and biopsy needle. Initial studies using the BioSentry hydrogel were conducted with a 19/20-gauge biopsy system [19,20]. One of the early BioSentry studies by Ahrar et al. demonstrated a reduced pneumothorax rate of 20.8% compared to 32.8% in controls. Similarly, chest tube insertion rates decreased to 8.2% vs. 20.8% in controls [20]. Another study by Grage et al. did not find a statistically significant difference in pneumothorax rates-31% in controls vs. 29% in the treatment group (p = 0.498). However, they observed a statistically significant difference in chest tube insertion rates, with 10% in controls vs. 2% in the treatment group (p = 0.032) [19]. In our study, we found a significant reduction in pneumothorax rates when using the sealant device, with an 18% rate in the treatment group vs. 26% in controls. Additionally, the BioSentry hydrogel reduced pneumothorax rates in "higher risk" patients with radiological evidence of emphysematous changes in the lung parenchyma. The chest tube insertion rates in the two initial studies ranged from 2-7% in the treatment group and 10-18% in the control group, while our cohort had an overall chest tube insertion rate of approximately 2% [19,20]. However, we did not find a significant difference in chest tube insertion rates between the treatment arm and the control group. The lack of a significant difference could be due to the low number of chest tubes inserted (7 out of 272) in our study. This suggests that our study might be underpowered to detect differences in chest tube insertion rates. Nonetheless, the difference in pneumothorax rates was evident despite being underpowered for chest tube insertion rates. Additionally, the lack of difference in chest tube insertion rates may be attributed to the significantly higher rates of severe emphysema, which could increase the baseline risk of symptomatic pneumothorax compared to patients without severe emphysema. Our pneumothorax rates were similar to those reported by Ahrar et al. and slightly lower than those in the study by Grage et al. [19,20]. Although sealant plugs generally reduce post-procedural complications, BioSentry has demonstrated superior performance in reducing complications, particularly in populations with emphysema.

Future studies can be directed toward a comparison of alternative agents for sealing pleural tracts. A recent publication by Zlevor et al. retrospectively analyzed 1,112 biopsies and concluded that pleural-parenchymal blood patch deployment upon removal of the introducer needle reduced post-procedural complication rates, including pneumothorax and subsequent chest tube insertion [21]. Other studies with similar aims have shown that using a sealant agent along the lung biopsy tract significantly reduces pneumothorax rates in patients undergoing CT-guided lung biopsy. Moreover, rates of chest tube placement and post-procedure hospital admission were also reduced [7-16, 19-22].

Limitations of our study include its retrospective nature, the smaller sample size, which made it difficult to detect differences in chest tube insertion rates, the fact that all biopsies were performed at a single institution, and the heterogeneous population, with a higher proportion of patients with emphysema in one group.

## Conclusions

Percutaneous CT-guided biopsy of pulmonary nodules using a 17/18-gauge coaxial system with a hydrogel plug pleural sealant device reduces the pneumothorax rate compared to patients who did not receive a pleural sealant. Although chest tube insertion rates were not significantly different between the BTS and no-sealant groups, this may be attributed to the significantly higher rates of severe emphysema, which increase the baseline risk of symptomatic pneumothorax compared to patients without severe emphysema. Further studies with larger sample sizes are needed to discern the differences in chest tube insertion rates between the BTS and no-sealant groups.
